# Knowledge, Attitudes, and Beliefs About Human Papillomavirus (HPV) Vaccination Among Puerto Rican Mothers and Daughters, 2010: A Qualitative Study

**DOI:** 10.5888/pcd11.140171

**Published:** 2014-12-04

**Authors:** María E. Fernández, Yen-Chi L. Le, Natalie Fernández-Espada, William A. Calo, Lara S. Savas, Camille Vélez, Angela Pattatucci Aragon, Vivian Colón-López

**Affiliations:** Author Affiliations: Yen-Chi L. Le, Natalie Fernández-Espada, Lara S. Savas, The University of Texas School of Public Health, Center for Health Promotion and Prevention Research, Houston, Texas; William A. Calo, University of North Carolina at Chapel Hill, Gillings School of Global Public Health, Chapel Hill, North Carolina; Camille Vélez, Angela Pattatucci Aragon, Vivian Colón-López, University of Puerto Rico, San Juan, Puerto Rico.

## Abstract

**Introduction:**

The incidence of human papillomavirus (HPV) infection and cervical cancer can be reduced by increasing vaccination for HPV. Yet vaccination uptake and completion of the 3-dose series remain low among Puerto Rican females. This study explored psychosocial factors associated with HPV vaccination uptake decisions among Puerto Rican mothers and daughters.

**Methods:**

We conducted 7 focus groups with young women aged 16 to 24 (n = 21) and their mothers (n = 9) to assess knowledge, attitudes, and beliefs related to cervical cancer, HPV, and HPV vaccination. We analyzed the focus group transcripts and identified themes by using a constant comparison method of qualitative data analysis and interpretation, guided by a grounded theory approach.

**Results:**

The analysis identified several emergent themes related to vaccine uptake: 1) low knowledge about cervical cancer, HPV, and the HPV vaccine; 2) inconsistent beliefs about susceptibility to HPV infection and cervical cancer; 3) vaccine effectiveness; 4) vaccine safety and side effects; 5) concerns that the vaccine promotes sexual disinhibition; and 6) availability of insurance coverage and overall cost of the vaccine.

**Conclusion:**

Our study found that adolescent girls and young women in Puerto Rico have low levels of knowledge about HPV and cervical cancer, low perceived susceptibility to HPV, and concerns about the safety and efficacy of the vaccine, and these factors may influence uptake and completion of HPV vaccination. Interventions are needed for both mothers and daughters that address these psychosocial factors and increase access to vaccination.

## Introduction

Hispanic women in the United States have higher rates of cervical cancer (1) than women in other racial/ethnic groups. In Puerto Rico, cervical cancer is the 6th most commonly diagnosed cancer among women (2). Two vaccines, Gardasil and Cervarix, prevent infections by high-risk human papillomavirus (HPV) types, which cause most cervical cancers (3). Gardasil, approved in 2006, is recommended for both young men and young women, and protects against HPV types 6, 11, 16, and 18; Cervarix, approved in 2009 for young women, protects against HPV types 16 and 18.

Rates of HPV vaccine initiation (51%) and completion (21%) are lower among girls aged 11 to 18 years in Puerto Rico (4) compared with young Hispanic women in the mainland United States (62.9% initiating and 59.3% completing the series). This disparity exists despite availability of free vaccination for children aged 11 to 18 years in Puerto Rico through several programs: Vaccines for Children (VFC), the Puerto Rican governmental health insurance plan (Mi Salud), and State Law no. 255, which mandates health insurance coverage of the HPV vaccine for girls and boys aged 11 to 18 years. Among US Hispanic women in the “catch-up” age range (19–26 years), HPV vaccination initiation rates (18.7%) are even lower than among adolescents (5). In Puerto Rico, programs for free vaccination do not cover women aged 19 to 26. Some, but not all, private insurance companies cover vaccination for women in the catch-up age group. Mi Salud participants, the uninsured, and those with medical insurance not covering the vaccination for this age group, can obtain it at a cost of $100 to $110 per vaccine dose at 6 vaccination centers across the island. Thus, access to vaccination among women aged 19 to 26 may be influenced by cost barriers.

Parental psychosocial factors that influence vaccination are knowledge (6–8), physician recommendation (9,10), and the belief that vaccination will protect daughters against genital warts and cervical cancer (6,10). Studies among women aged 18 to 26 (11,12) and 19 to 26 (13) report that knowledge and awareness of HPV (11), beliefs and attitudes about HPV vaccine (13), access to transportation (12), and prioritizing health over other daily responsibilities (12) are associated with vaccine uptake.

Research among Hispanic mothers and daughters in the United States (6,14) revealed key predictors of vaccine uptake, but information about psychosocial factors influencing vaccination among women in Puerto Rico is not available. Although young women aged 19 to 26 may have more autonomy in HPV vaccine decision-making than do underage girls, they may still rely on parental input or financial support for vaccination. The purpose of this study was to explore factors influencing HPV vaccine initiation decisions by mothers and daughters in Puerto Rico.

## Methods

Recruitment announcements were posted throughout hospitals and clinics at San Juan Medical Center and in communities identified through our outreach network (15). We recruited young women aged 16 to 26 and mothers of daughters in the same age range. Although recruitment efforts targeted fathers and mothers, except for one father, all participants were mothers. We removed the father’s data from the analysis for consistency and analyzed only data from mothers. Other eligibility criteria included daughters’ knowledge of their own HPV vaccination status, mothers’ knowledge of their daughters’ HPV vaccination status and no history of cervical cancer for either group. Among 44 eligible mothers and young women, 30 (68%) agreed to participate. Participants were assigned to a daughter- or mother-focus group and were stratified by HPV vaccination status (daughter had at least one HPV vaccine, and those never vaccinated). All mothers had daughters that participated in the young women’s groups. However, some young women did not have their mother in the parents’ groups. The institutional review boards of the University of Texas Health Science Center and the University of Puerto Rico Medical Sciences Campus approved the study protocol.

We conducted 7 focus groups during April through June 2010: 4 with young women (n = 21) and 3 with mothers (n = 9). Three groups consisted of unvaccinated daughters (n = 16) and one group consisted of vaccinated daughters (n = 5). Two parental groups had unvaccinated daughters (n = 6) and one group had vaccinated daughters (n = 3). Group discussions were held at an academic institution in the San Juan Medical Center. The study team adapted an existing interview guide (Box 1) from a previous study (6). The facilitator described the study and obtained informed consent. Women younger than 18 years provided a parent-signed informed consent and their assent to participate. All participants completed a short demographic questionnaire. Female facilitators conducted the 1-hour focus groups in Spanish. Following the discussion, participants received educational materials about HPV and cervical cancer, along with $20 as compensation. Focus groups were audio-recorded, transcribed, and entered into Atlas.ti (ATLAS.ti GmbH) for analysis.

**Box 1. Interview Questions From Focus Groups Conducted in Puerto Rico With Mothers and Daughters About Their Knowledge, Attitudes, and Beliefs About Human Papillomavirus (HPV) Vaccination, 2010**

**Table:** 

Topic	Question
Awareness and knowledge of cervical cancer	What do you think when you hear cervical cancer? What do you know about cervical cancer? What causes cervical cancer? Who can have cervical cancer? Do you think there is a possibility your daughter may have cervical cancer in the future? Why? Why not? (Daughters’ groups only) Do you think there is a possibility you may have cervical cancer in the future? Why? Why not?
Awareness and knowledge of HPV and the HPV vaccine	Have you heard about the human papilloma virus (HPV)? Where did you hear about it? Who could get HPV? (Daughters’ groups only) Do you think you could get infected with HPV? Have you heard about the HPV vaccine? What do you know about the HPV vaccine?
Attitudes toward the HPV vaccine	What do you think about vaccinating your daughter? Why? Why not? What reasons do you have for vaccinating or not vaccinating your daughter? What concerns would you have about vaccinating your daughter? What information would you need to make a decision? (Daughters’ groups only) What do you think about getting vaccinated? (Daughters’ groups only) What do you think would be the benefits of getting vaccinated? (Daughters’ groups only) What concerns would you have about getting the HPV vaccine?

The lead author and 3 coders read all transcripts and began coding by using a constant comparison method, consistent with a grounded theory approach (16). Coders read and coded the same transcript, and then discussed identified themes and produced a base set of codes to use for the remaining transcripts. The analysis team met biweekly to discuss new themes and to come to consensus on new codes and coding discrepancies.

## Results

The analysis of the focus group transcripts allowed us to identify several emergent themes related to vaccine uptake: 1) low knowledge about cervical cancer, HPV, and the HPV vaccine; 2) inconsistent beliefs about susceptibility to HPV infection and cervical cancer; 3) misconceptions about vaccine effectiveness; 4) concern about vaccine safety and side effects; 5) concern that the vaccine promotes sexual disinhibition; and 6) lack of insurance coverage and overall cost of the vaccine (Box 2). Daughters’ mean age was 20.4 years (standard deviation [SD], 3.8 y) and mothers’ was 47.9 years (SD, 5.5 y). Most daughters had completed 1 or more years of college education (67%); 86% of mothers had a bachelor’s or advanced degree. All daughters had health insurance. Slightly more than half (55%) of participants reported a family income below $40,000 annually.

**Box 2. Additional Quotes From Focus Groups Conducted in Puerto Rico With Mothers and Daughters About Their Knowledge, Attitudes, and Beliefs About Human Papillomavirus (HPV) Vaccination, 2010**

**Table:** 

Theme	Example Quote	Group
Low knowledge about cervical cancer and its causes	“I know it [cervical cancer] develops in the cervix, but I don’t know anything else about it.”	Vaccinated daughter
	“I know it’s caused by the human papillomavirus, but I don’t know why.”	Unvaccinated daughter
	Who can get cervical cancer? “People that don’t have good hygiene.”	Unvaccinated daughter
	“I don’t know how to explain it [cervical cancer] because I don’t know much about it.”	Mother of vaccinated daughter
	“I think that once they tell you that [cervical cancer diagnosis] they have to get everything out before it spreads to other areas.”	Mother of unvaccinated daughter
	“I have heard about it [HPV], but I don’t know what it is.”	Vaccinated daughter
	“I was once at a store . . . when I went to try the bra the sales clerk told me ‘No, because there is a new virus, HPV, and you can’t [try the bra] because it’s contagious [HPV].’”	Unvaccinated daughter
	“I have heard that like other cancers its [cervical cancer] origin is not known. I know some are treatable if found early, even cervical cancer, but I don’t have too much information about HPV.”	Mother of vaccinated daughter
	What is the most important factor that influenced your decision of not vaccinating your daughter? “Complete lack of knowledge. I don’t know the factors, the pros and cons.”	Mother of unvaccinated daughter
	“Gardasil can prevent infection, but it has a target . . . I understand it’s for 13 to 20 years old.”	Mother of unvaccinated daughter
Concerns about vaccine efficacy	“They are still doing the studies and I might be somewhat protected now but in a few years who knows.”	Vaccinated daughter
	“I don’t know how long the doses last. Do I have to get another vaccine [booster] again?”	Unvaccinated daughter
	“So I’m not sure if they [daughters] can get it if they have had sex already or should they still get it if they are still in that age range?”	Mother of unvaccinated daughter
Concerns about vaccine safety and side effects	“Everything has a risk, every vaccine has side effects.”	Vaccinated daughter
	“It’s new and some things haven’t come out yet, like side effects, so I wait years before trying something new.”	Unvaccinated daughter
	“At that moment I was worried that something would happen to her, she would [be] left sterile or get another illness.”	Mother of vaccinated daughter
	“And what about if this vaccine causes her sterility in the future?”	Mother of unvaccinated daughter
Concerns about the vaccine promoting sexual disinhibition	“She [mother] thinks that if she vaccinates her daughter or she tells her [about the vaccine] it would be giving her the green light, ‘you can do it [sex].’”	Vaccinated daughter
	“Now she will become sexually active because she is vaccinated.”	Mother of unvaccinated daughter
Lack of insurance coverage and overall cost of the vaccine	“It’s hard because you say it is one hundred dollars the first dose, ok we get the hundred dollars. But for this next date you need to have another hundred dollars so some people may say ‘I have to wait and save [money].’”	Vaccinated daughter
	“I went to the clinic to find out when I was due for vaccines, but my mom didn’t want me to get this one because it was too expensive.”	Unvaccinated daughter
	“My kids’ pediatrician offered me the vaccine for them, but I didn’t get it because it’s three hundred dollars each dose and the medical plan doesn’t cover it.”	Mother of unvaccinated daughter

### Low knowledge about cervical cancer and HPV

**Young women.** Few participants from either group said that cervical cancer is caused by HPV, multiple sexual partners, or lack of routine screening. Unvaccinated women had little knowledge about HPV or the vaccine. They said that cervical cancer is caused by genetic predisposition, use of birth control pills, lack of hygiene, poor diet, and lack of physical activity. Misconceptions included that HPV could be transmitted by sharing a bathing suit or soap. Most participants had not heard about the vaccine and could not describe vaccination guidelines. One participant noted that women do not receive information from their gynecologists about the vaccine.

**Mothers.** Most mothers had difficulty explaining what cervical cancer is and suggested several causes, including HPV, genetic predisposition, promiscuity, and early initiation of sexual activity. Some mothers of unvaccinated girls also said that cervical cancer is fatal “like HIV.” With one exception, all mothers said that regular Papanicolaou (Pap) tests could detect cervical cancer. Only one mother knew that HPV is sexually transmitted. Most mothers of unvaccinated daughters believed that HPV affects only women. Many mothers expressed not knowing enough about the vaccine, including vaccination guidelines, and most wondered whether girls could still get the vaccine after they had initiated sexual activity.

### Susceptibility to cervical cancer and HPV infection

**Young women.** Regardless of their vaccination status, most young women said “anyone is at risk of [cervical] cancer,” including themselves. When asked about HPV, some vaccinated daughters indicated that their risk for getting HPV is low because “they got the vaccine.” Others still felt at risk because the vaccine “only covers a few types of HPV.” Unvaccinated young women noted “you are never exempt.” A younger unvaccinated participant indicated not feeling at risk for HPV because she is not sexually active.

**Mothers.** When mothers were asked if they felt their daughters were at risk for cervical cancer, some indicated that they “don’t think about this.” Mothers with unvaccinated daughters were more likely to discuss reasons why their daughters were not at risk, including their belief that they were not sexually active. One mother highlighted the distinction between what she thought other girls were doing (being sexually active at a young age) in contrast to her daughter. “It’s about lifestyle; I think that girls can be at risk if they are sexually active. I’m a high school teacher and I know that more than 50% are sexually active; my three daughters are not. We have talked about it with openness and honesty . . . our family values are very different from those of the girls I teach.”

### Concerns about vaccine efficacy

**Young women.** Some young women were not sure whether the vaccine would be effective if they had already had sexual relations: “I think that people our age would say ‘it’s not useful for me, I’m already sexually active,’ since the vaccine is advertised for people who haven’t yet.” Participants also expressed doubts about the [HPV] vaccine’s effectiveness in general: “I haven’t heard about how effective the vaccine is . . . or about the probability of decreasing my risk of infection if I vaccinate now.” Other young women wondered how long the vaccine’s protection lasted. Others shared this confusion and concern that they may not be protected in the future.

**Mothers.** Some mothers expressed hesitation about vaccinating their daughters and alluded to the unknowns about effectiveness. One mother said that she vaccinated her children with all the required vaccines to enter school but she is “not convinced” of the efficacy of the HPV vaccine. Others said that if it is so important, the government should “take action to educate the community.” Their perception was that since the HPV vaccine was not publicized much, it must not be important or efficacious.

### Concerns about vaccine safety

**Young women.** Most vaccinated young women were not concerned about the vaccine’s potential side effects. One participant said that “everything has risks and I think the benefits outweigh the risks.” Only one young woman expressed fear because she had read an article highlighting some deaths among vaccinated girls. Unvaccinated women mentioned several concerns including acquiring another illness as an effect of the vaccine and unknown long-term side effects: “I would have to know what the risks are so I won’t be preventing one illness, but getting other [illnesses].” Another participant responded, “It’s new and some things haven’t come out yet, like side effects, so I wait years before trying something new.”

**Mothers.** Mothers of vaccinated girls were concerned that the vaccine would leave daughters sterile or cause another illness and that the vaccine was still being researched so daughters would be “guinea pigs.” Mothers with unvaccinated daughters had more concerns than those who had vaccinated daughters. Concerns were unknown side effects, unknown level of safety, and fear that the vaccine has not been researched enough. One participant mentioned: “I have doubts, I talk with my cousin and she says that side effects are still unknown, that it is not safe, and that we have to wait.”

### Concerns about the vaccine promoting sexual disinhibition

**Young women.** In general, daughters did not agree with their mothers’ concern that getting vaccinated was giving them permission to be sexually active. They said “it’s not giving permission; it’s protecting your child.” Young women were more likely to agree that younger girls (even aged 9) should be vaccinated to be protected before becoming sexually active. They also noted that if mothers don’t want to vaccinate daughters, it should really be the daughter’s decision. Most said 16 or 17 years of age would be an appropriate age for girls to make their own decision about vaccination.

**Mothers.** Some mothers worried that HPV vaccination would send daughters the message that it was “ok” to have sex and if daughters believed they were protected from HPV, they would feel free to have sex. One participant said: “I don’t know to what extent a girl that’s 9 years old might assume ‘oh I’m protected; now I can have sex’. So you really have to be careful at this age.” Most mothers across groups believed that the appropriate age to vaccinate daughters is when they enter high school or college.

### Overall cost of the vaccine and availability of insurance coverage

**Young women.** Young, unvaccinated women highlighted financial barriers and noted that “although many people want to obtain the vaccine, they can’t pay for it” or “we would have to pay a high deductible.” Indeed, a vaccinated participant responded that having access to free vaccination through health insurance was an important factor in her vaccination: “[I] did not have any inconvenience; the health insurance covered the full price of the vaccine.”

**Mothers.** Mothers noted that they couldn’t afford the vaccine. One mother said: “the pediatrician recommended the vaccine for my kids, but I didn’t vaccinate them because of the cost; the health insurance does not cover it.” Another mother of a vaccinated participant stated that “without insurance covering the HPV vaccine she could not afford the vaccine.”

## Discussion

This study explored psychosocial factors influencing the HPV vaccination decisions of mothers and daughters living in Puerto Rico. Although several of our findings are similar to those of other studies (6,14,17), we identified unique factors and possible differences in the degree of importance for some of the common ones. For example, despite high levels of education and income among our participants, their knowledge about HPV and HPV vaccination was low, especially among mothers. Additionally, their concerns about vaccine efficacy and, to some extent, safety seemed to be associated with their perception that the HPV vaccine had neither been promoted in Puerto Rico nor recommended by their providers.

Provider recommendation, a factor that influences vaccine decisions (17,18), may be of particular relevance in Puerto Rico. Studies on role preference in health care decision-making show that Hispanic patients often prefer that providers take the lead in making health care decisions (19). This situation may translate into less willingness to ask a provider to vaccinate their daughters and less willingness to engage in health-related behaviors without explicit recommendations from providers.

Mothers expressed reluctance to vaccinate younger daughters and did not understand the benefit of HPV vaccination before initiation of sexual activity. Maternal concerns that HPV vaccination would promote sexual disinhibition have been reported elsewhere (20,21). In a study with Latina parents in 3 different regions of the United States, concern about HPV vaccination promoting sexual disinhibition ranged from 11% in Los Angeles to 60% in Seattle (17). Prior work also has shown that this concern is more likely among older parents and those reporting conservative views (4). However, increased knowledge about the HPV vaccine decreases the likelihood of having this belief (21).

Mothers also expressed concerns regarding vaccine efficacy and safety. Other studies with Hispanic populations reported similar concerns (6,8,10). Low immunization rates overall in Puerto Rico (measles, mumps, and rubella [MMR], 85.2%; diphtheria, tetanus, and pertussis [DTaP], 81.9%; varicella [chickenpox], 88.7%) (4) may be related to concerns about the overall safety of vaccines and should be considered a contextual factor in mothers’ reluctance to have their daughters vaccinated for HPV. Although some daughters, particularly unvaccinated ones, had concerns, they generally had a better understanding of the need for vaccination and recognized the benefits of following recommended HPV vaccination guidelines.

This study has some limitations. Given the nature of qualitative inquiry, results are not generalizable to all women in Puerto Rico. Additionally, participants were of higher socioeconomic status than the general population in Puerto Rico. For 2008 through 2012, the annual median household income in Puerto Rico was $19,515 (22), whereas in our study 75% of the young women and all of the mothers had a yearly income of $20,000 or higher. Although 67% of the young women in the groups had at least 1 year or more of college education, only 27.8% of young people aged 18 to 24 in Puerto Rico had completed high school. Nevertheless, even with higher education and income levels, knowledge of HPV and the vaccine was still low. Young women and mothers may now have different or additional concerns than participants in our study in 2010. However, since HPV vaccination rates are still low, probably for many of the same reasons (4), we believe our study findings provide a valuable contribution to the factors associated with low levels of HPV vaccination in Puerto Rico.

Our findings are consistent with those of other studies, yet some factors varied in the degree of importance for Puerto Ricans. Increasing the level of HPV vaccination may not necessitate new interventions that are vastly different from those developed for other groups. Instead, moderate adaptation of interventions may make them more relevant for Puerto Rican women. For example, given mothers’ doubts about vaccine efficacy due to low recommendation and promotion of the vaccine, messages focusing on vaccine efficacy, recommendations from providers, and increased promotion of the HPV vaccine may be warranted. Additionally, because mothers seem to be involved in the vaccine decision for economic or cultural reasons, interventions focusing on communication between mothers and daughters about HPV may increase vaccination. Modifying promotional materials to match them with Puerto Rican culture may also be warranted. A recent study (15) that explored the role of ethnic identity on attitudes toward HPV vaccine advertising noted that Puerto Rican participants did not identify with many of the HPV vaccine campaigns because the actors’ facial characteristics and language were not Puerto Rican. Participants also did not feel at the same risk levels as the individuals portrayed in the advertisements (15).

Our study is the first to provide insight into common psychosocial barriers affecting HPV vaccination in Puerto Rico and underscores the need for increased education about HPV vaccination, provider recommendations, and access to low-cost vaccines, similar to findings reported from other studies (17,23–26). Future interventions should address knowledge regarding HPV vaccination effectiveness and safety, the rationale behind HPV vaccination for young age groups, and the potential benefits for vaccinating young women in the catch-up age range. The Community Cancer Control Outreach Program, the Cancer Center Coalition at the University of Puerto Rico, and the Puerto Rico Comprehensive Cancer Center are implementing initiatives that translate such findings into messages and strategies to promote HPV vaccination throughout Puerto Rico.
